# Differential responses of SARS-CoV-2 variants to environmental drivers during their selective sweeps

**DOI:** 10.1038/s41598-024-64044-1

**Published:** 2024-06-10

**Authors:** Thomas P. Smith, Swapnil Mishra, Ilaria Dorigatti, Mahika K. Dixit, Michael Tristem, William D. Pearse

**Affiliations:** 1 Georgina Mace Centre for the Living Planet, Department of Life Sciences, Imperial College London, Silwood Park, Ascot, Berkshire, SL5 7PY UK; 2 Saw Swee Hock School of Public Health and Institute of Data Science, National University of Singapore and National University Health System, 12 Science Dr 2, Singapore, 117549 Singapore; 3 MRC Centre for Global Infectious Disease Analysis, School of Public Health, Imperial College London, 90 Wood Lane, London, W12 OBZ UK

**Keywords:** SARS-CoV-2, Transmission, Variant, Climate, Temperature, Ecological epidemiology, Infectious diseases

## Abstract

Previous work has shown that environmental variables affect SARS-CoV-2 transmission, but it is unclear whether different strains show similar environmental responses. Here we leverage genetic data on the transmission of three (Alpha, Delta and Omicron BA.1) variants of SARS-CoV-2 throughout England, to unpick the roles that climate and public-health interventions play in the circulation of this virus. We find evidence for enhanced transmission of the virus in colder conditions in the first variant selective sweep (of Alpha, in winter), but limited evidence of an impact of climate in either the second (of Delta, in the summer, when vaccines were prevalent) or third sweep (of Omicron, in the winter, during a successful booster-vaccination campaign). We argue that the results for Alpha are to be expected if the impact of climate is non-linear: we find evidence of an asymptotic impact of temperature on the alpha variant transmission rate. That is, at lower temperatures, the influence of temperature on transmission is much higher than at warmer temperatures. As with the initial spread of SARS-CoV-2, however, the overwhelming majority of variation in disease transmission is explained by the intrinsic biology of the virus and public-health mitigation measures. Specifically, when vaccination rates are high, a major driver of the spread of a new variant is it’s ability to evade immunity, and any climate effects are secondary (as evidenced for Delta and Omicron). Climate alone cannot describe the transmission dynamics of emerging SARS-CoV-2 variants.

## Introduction

SARS-CoV-2, the causative agent of COVID-19, has had a major impact on global health, with over 750 million confirmed cases and over 6.9 million deaths, as of 17-05-2023^1^. Quantifying the factors driving the transmission of SARS-CoV-2 is an ongoing and critical global challenge^2^. In particular, many studies have compared the effects of control measures to those of environmental, socioeconomic, and demographic factors, as well as climate factors thought to drive a seasonal response^2–6^. Understanding these factors and their interactions is critical to successfully implementing strategies to control this pandemic^2^. The general consensus across this vast body of work is that the implementation of non-pharmaceutical interventions (NPIs) and vaccines is the major driver of SARS-CoV-2 transmission rates, but that climate seasonality^2,4–7^, population density^4–6^, and socioeconomic factors^8^ can be significant secondary predictors of transmission, particularly in the absence of NPIs^6^ and immunity^2^.

However, the overall landscape of transmission has changed dramatically since the initial phases of the pandemic, as new SARS-CoV-2 variants have repeatedly emerged and in most cases swept away previous variants to become the new dominant strain in a given place^9^. These rapid turnovers from one dominant strain to another following the logistic equation can be interpreted as “selective sweeps”^10^. Furthermore, the types and effectiveness of NPIs have also changed over the course of the pandemic. In the UK for example, mobility restrictions varied from full ‘lockdowns’ early in the pandemic, to a later system of tiered restrictions which varied dynamically across time and space^11^. Finally, since the end of 2020, vaccination programs have been rolled out, further altering the environment within which transmission occurs. All of this leads to a situation in which our understanding of the impacts of environmental drivers must be reassessed within the context of newly emerging viral lineages, varying interventions and immunity. To date, the influence of climate on the spread of new variants, through selective sweeps, has not been assessed.

In late 2020, approximately 9 months after SARS-CoV-2 first entered the UK, lineage B.1.1.7^12^ (named ‘Alpha’ by the World Health Organisation) rapidly spread through the UK population to become the dominant strain across the country. Subsequently in Spring 2021, lineage B.1.617.2 (‘Delta’) rapidly spread in a new selective sweep to become the dominant variant in the UK^13^. Later, lineage B.1.1.529.1 (‘Omicron’ BA.1) spread even more rapidly through the UK than previous variants^14^. The first case of Omicron in England was reported in late November 2021, and by early January 2022 it had spread to account for almost all new cases in the country^15^. Such selective sweeps ultimately occur due to the higher reproduction number, *R*, of the sweeping variant in comparison to other circulating strains^16^. Differences in *R* can depend upon a range of factors, such as the intrinsic transmissibility of different variants, or evasion of prior immunity and interactions with NPIs^13^. Whether the response of variants to environmental factors could also influence the outcome of competition is currently unknown. Differences in the spike protein of the Alpha, Delta and Omicron variants cause them to produce different results in a common diagnostic PCR test used in the UK (Alpha and Omicron are S-gene negative, Delta is S-gene positive^13,16^). Using data derived from these test results alongside models of transmission rate, it is possible to reconstruct the transmission rates attributable to the background strain(s) and the newly invading variant^16^. A large body of such data has been collected in the UK, allowing the dynamics of SARS-CoV-2 transmission to be investigated during these three selective sweeps.

Here, by combining previous transmission rate estimates for the Alpha variant^16^ with new estimates for the Delta and Omicron variants, we investigate transmission of these variants during their respective selective sweeps across England. We quantify the variation attributable to NPIs, vaccination rates, population density, temporal, and spatial effects, and then compare the resulting residual variation in transmission rates to climate variables. We ask whether there are differences in the environmental responses of these variants, and investigate whether any differences in response may have facilitated their respective spread through England.

## Results

**Figure 1:**
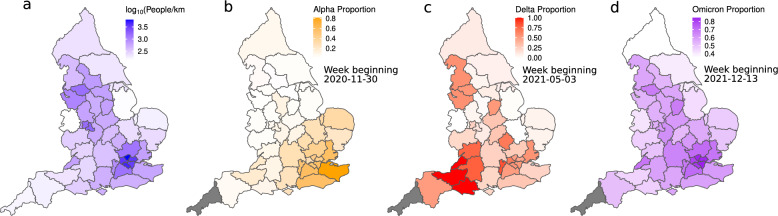
Spatial transmission dynamics during variant selective sweeps. (**a**) Population density map of England; transmission is expected to be higher where there is greater population density^4–6^. (**b**, **c** and **d**) Spatial dynamics of Alpha, Delta and Omicron spread respectively; plots show the proportion of positive cases identified as the spreading variant. Alpha’s spread in England began in the South–East (hence its early denotation as the “Kent variant”^19^), Delta first became the dominant strain in the South–West, whereas Omicron first became dominant in London and the North–West. Notably, the Omicron variant first dominated many of the most population dense areas. Cornwall (grey) was omitted from our analyses as this region generally had too few deaths for accurate $R_t$ estimates. Maps produced in R, version 4.1.2^20^, based on geometry for the UK National Health Service’s “Sustainability and Transformation Plan” regions (STPs) obtained from the Office for National Statistics^21^.

**Figure 2:**
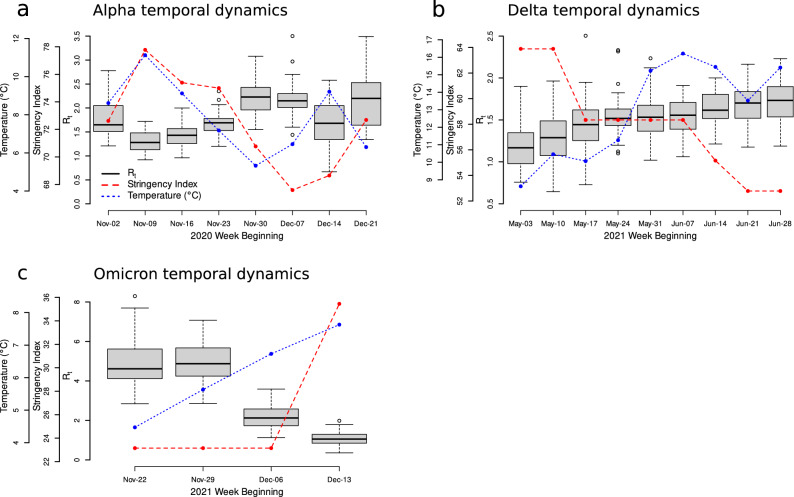
Temporal transmission dynamics during selective sweeps of Alpha (**a**), Delta (**b**) and Omicron (**c**). Box-plots of transmission rates from each region in England on a weekly basis (open circles are outliers), with overlaid points and lines showing average (mean) temperature for that week (blue) and the stringency index (red; a measure of non-pharmaceutical intervention strength^22^). Previous work predicts negative relationships between *R* and both temperature and NPI stringency^6^, *i.e.* when temperature and NPI strength are high, $R_t$ is expected to be low. During Alpha’s spread through England, transmission rose and fell roughly in sync with changes in temperature and NPI strength ($R_t$ increasing when temperature and NPI strength drop, and vice-versa). During Delta’s sweep, transmission increased as NPIs were lessened, despite increases in temperature through time. During Omicron’s selective sweep, transmission reduced as temperatures increased, however there was also an increase in NPI stringency towards the end of the Omicron sweep. Note there are different vertical axis scales in each subplot - these figures are for visualisation of trends only.

There are differences in the spatial and temporal dynamics of each variant’s selective sweep through England (Figs. 1 and 2). The Alpha variant was first detected in South–East England and this is the first area in which it became the dominant strain (Fig. 1b). By comparison, the Delta variant (which likely first arose outside of the UK^17,18^) became the dominant strain in the South–West before sweeping through the rest of England (Fig. 1c). The spread of Omicron had different spatial dynamics again, becoming the dominant strain first in London and the North–West. We also show variation in population density across England (Fig. 1a), an important non-climate factor in SARS-CoV-2 transmission rates^4–6^. Areas where Omicron swept to dominance first tended to be those with the highest population densities.

We investigated the temporal dynamics of transmission of each variant during it’s selective sweep; Alpha between the weeks beginning 2nd November and 21st December 2020; Delta between the weeks beginning 3rd May and 28th June 2021; Omicron between the weeks beginning 22nd November and 13th December 2021. Alpha tended to show higher $R_t$ during its winter sweep, than Delta did during its spring/summer sweep (Alpha $R_t$ mean = 1.80; Delta $R_t$ mean = 1.51; Welch’s t-test $t_{522.5} = 9.06$, $p < 0.001$; Fig. 2a). However, there was also much greater variation in $R_t$ across space and through time during the Alpha sweep than during the Delta sweep (Alpha $R_t$ SD = 0.496; Delta $R_t$ SD = 0.298; Levene’s test for homogeneity of variance $F_{1818} = 167.27$, $p < 0.001$; Fig. 2). By comparison, Omicron showed much higher $R_t$ at the start of its sweep than either the Alpha or Delta variants during any points of their selective sweeps (Fig. 2c). As the Alpha variant swept through England, its transmission rate oscillated through time (Fig. 2a). These oscillations are inverse to the fluctuations in temperature through time (i.e. $R_t$ tended to increase when temperature decreased, and vice-versa), however there is also a similarly fluctuating temporal effect of changes in NPI strength. By comparison, the transmission rate of the Delta variant increased gradually through time as it swept through England (Fig. 2b). During the Delta sweep, NPIs were gradually relaxed, whilst temperature tended to increase. There are many fewer time-points to analyse during the Omicron sweep due to it’s rapid rise to dominance, however a similar overall pattern is seen to that of the Alpha sweep; $R_t$ decreases as temperature and NPI strength increase (Fig. 2c).

We first eliminate variation in $R_t$ attributable to known non-climate spatial and temporal factors, leaving behind a residual variation in $R_t$ within which we can investigate climate predictors of transmission (see methods). For the Alpha variant sweep, 31% of the variation in $R_t$ can be explained by NPI stringency, population density, spatial and temporal effects (Table 1). For the Delta variant, 46% of the variation in $R_t$ is attributable to these factors, plus the proportion of the population vaccinated in each region through time (Table 1). For Omicron, almost all of the variation in $R_t$ is attributable to these non-climate factors (82.6%, Table 1).

**Table 1:** Variation in $R_t$ **attributable to non-climate factors.**

	$r^2$ (%)	*F*-statistic	DF	Residual SE	*p*-value
Alpha	30.7	2.86	44 and 283	0.44	$< 0.001$
Delta	45.6	6.01	45 and 323	0.23	$< 0.001$
Omicron	82.6	12.41	44 and 115	0.95	$< 0.001$

Here we provide the regression statistics obtained in the first stage of the analysis, to remove variation in $R_t$ unrelated to climate. More variation in $R_t$ can be explained by these non-climate factors for sequential variant sweep (higher model $r^2$ for Omicron than Delta, which is higher than Alpha).

**Figure 3:**
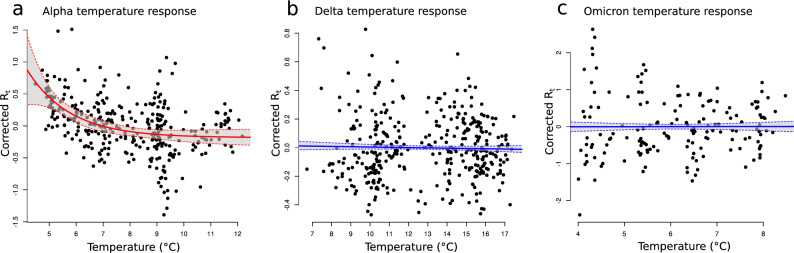
Impacts of temperature on variant transmission rates. Here we plot the residuals from linear regressions of $R_t$ versus all of the non-climate spatial and temporal predictors against temperature, for the Alpha (**a**), Delta (**b**) and Omicron (**c**) variants respectively. After accounting for the non-climate-related variation in $R_t$, we find that temperature had a significant impact on transmission rates during the Alpha variant sweep and, whilst a linear regression had a significant negative slope (supplementary figure 4), this is better described by an asymptotic model (red curve, likelihood-ratio test $p < 0.001$). We find no significant impact of temperature during either the Delta or Omicron variant sweeps (linear models, blue).

We then regress the residuals from these initial models against the potential climate drivers to understand their influence upon the remaining, unaccounted for variation in $R_t$ (see methods). We find that temperature, UV and precipitation all account for significant proportions of the residual variation in $R_t$ during the Alpha variant sweep, with temperature having the strongest effect (supplementary table S1). As temperature was the strongest predictor, we investigate this further and find that variation in $R_t$ due to temperature is better described as an asymptotic function than a linear function (likelihood-ratio test $p < 0.001$; Fig. 3a; supplementary table S4). By comparison, we find no strong effects of any climate predictors on the residual variation in $R_t$ during either the Delta or Omicron sweeps and for neither can any relationship with temperature be described by either an asymptotic curve or linear model (supplementary tables S2, S3 and S4, Fig. 3b and c).

## Discussion

Our study is the first to unpick and compare the role of climate in SARS-CoV-2 transmission during the selective sweeps of three new variants. We find strong evidence for a climate-driven response of SARS-CoV-2 Alpha variant transmission during its spread through England, however we find no response to climate factors for either the Delta or Omicron variants during their spread. These results have important implications for understanding when and where climate may enhance the progression of new variant sweeps, a critical factor in the progression of the worldwide pandemic as new, highly-transmissible variants continue to arise, particularly sub-lineages of Omicron^23,24^.

We tested the impacts of multiple climate variables in our regression analyses and found significant impacts of temperature, UV and precipitation on the transmission rate of the Alpha variant. However, there is difficulty disentangling the effects of multiple climate variables modelled together, due to the multiple collinearities amongst them (supplementary figures S1-S3). Therefore, as previously^6^, we focused on temperature as the strongest predictor for corrected $R_t$ (the largest coefficient with scaled variables). Other work has suggested that climate-related transmission drivers may act non-linearly, *i.e.* how changes in climate drive transmission may depend upon the specific state of the climate in any given place^4,5,25^. To allow for this, and after visualisation of the temperature vs corrected Alpha $R_t$ response, we fitted an asymptotic response curve alongside a basic linear response. We found that the non-linear asymptotic model best explained how the Alpha variant transmission rate varied with temperature.

That changes in climate can have differing effects on SARS-CoV-2 transmission at different times has important implications when considering the dynamics of selective sweeps of new variants. Our finding of a non-linear response of temperature during the Alpha variant sweep implies that when the climate is generally cold, further reductions in temperature lead to increased SARS-CoV-2 transmission rates. However, when the climate is milder, small fluctuations in temperature have little impact on transmission rates. In the context of emerging variants, colder temperatures are likely to increase the rate of transmission of a more transmissible variant, allowing it to sweep through the population in less time. Indeed, while Delta is suggested to have a greater intrinsic transmission rate than Alpha^13,16^, Alpha tended to show a higher transmission rate during its (winter) sweep across England than was shown by Delta during its (spring/summer) sweep. This was despite more stringent NPIs also being in place during the Alpha selective sweep compared to the Delta sweep. However, Omicron also showed no significant climate response despite also sweeping across England in winter during even colder conditions.

That we found no effect of climate during the Delta and Omicron sweeps may in part be due to our ability to explain the non-climate related variation in $R_t$. After introduction to England, the Delta variant swept aside all others to rapidly become the dominant strain^13^. This was not simply due to travel-related import of the new variant, but sustained community transmission, with Delta proving significantly more transmissible than previous lineages^13^. The $R_t$ of Delta continually increased during its sweep through England (Fig. 2b), despite England already having a high level of immunity across the population at this time due to vaccination roll-outs^13^. Potentially, the Delta variant’s transmission advantage over other strains may therefore have been influenced by an increased immune evasion^17,18^. Similarly, after its arrival in England, the Omicron variant very swiftly swept aside the Delta variant to become essentially the only strain circulating^15^. The swiftness of this spread was also likely due to its escape from vaccine-induced immunity^15,26^. A difference in the models used here to extract the non-climate related variance was the inclusion of vaccination rates during the Delta and Omicron sweeps (vaccines were not yet available during the Alpha variant sweep). Therefore, the inclusion of this extra factor allows the models to explain more of the non-climate related variance (particularly as both Delta and Omicron’s sweeps were driven in part by immunity evasion), leaving less to be potentially explained by climate. Indeed, for the Omicron variant sweep, almost all variation in $R_t$ could be explained by non-climate spatial and temporal effects (83% of variation). Essentially, the greater the effect of non-climate factors on transmission, the comparatively lesser the effect that climate may have on transmission of a particular variant during a selective sweep. This is similar to previous findings that when the strength of NPIs is high, climate has comparatively little effect on transmission rates^6,27^. Therefore, while climate may increase overall transmission rates in winter compared to summer, other factors, such as evasion of vaccine-induced immunity, are likely to be more important drivers of the spread of new variants.

While we don’t explicitly compare the differences in importance of each climate versus non-climate transmission factor here, we can assume from previous work that non-pharmaceutical interventions and immunity are likely the two major extrinsic drivers of transmission rate^2,6,27,28^. Indeed, far greater variance in $R_t$ could be explained by non-climate climate factors than climate factors for all three selective sweeps. Our methodology therefore first removed the residual variation explained by non-climate factors, in order to better understand the influence of climate variables upon $R_t$. It is possible, therefore, that our approach down-weights the importance of climate and weather, which is particularly problematic as NPIs are often correlated with temperature (see Fig. 2), but given the clear role that NPIs play, being statistically conservative is the correct approach. However, this also means that we may not necessarily be able to generalise beyond our study period, if climate effects are stronger in the absence of NPIs^2,6^. During our study period, NPIs were generally high, however at the time of writing, NPIs are generally either much less severe, or not in use in many places. Amongst non-climate drivers, population density has been shown to be important in explaining differences in $R_t$ between different regions^4–6^. It has been suggested that the population-weighted population density, or the “lived” population density (essentially, the average density that a person in the region experiences, rather than the average density of the region itself), may be a more appropriate measure than the raw population density when investigating large spatial areas, as this is more epidemiologically relevant^29^. However, England’s STP areas are comparatively small, negating this problem, and significant effects of population density have been found even when averaged across much larger spatial areas^6^. A potential drawback of using these data from England, is that they cover a comparatively narrower range of climate than have been tested in previous studies^2,4–6^. However, here we are investigating not just the response of SARS-CoV-2 to climate, but the responses of specific variants during their selective sweeps, and we know of no other datasets from which the $R_t$ of specific variants can be unpicked to the resolution here. Therefore, while greater spatial variation in temperature may have allowed us to draw stronger conclusions, it was not feasible for this study.

Overall, we have shown that climate can influence the selective sweeps of new SARS-CoV-2 variants in differing ways. Specifically, we found that during the Alpha variant sweep, comparatively little variation in $R_t$ could be explained by non-climate factors, however a significant proportion of the residual variation could be explained by climate, with temperature in particular displaying a strong non-linear relationship with $R_t$. In comparison, proportionally more variation in $R_t$ during both the Delta and Omicron sweeps could be explained by non-climate factors, and climate factors were unable to explain any significant proportion of the residual variation. Therefore, although climate may affect the overall $R_t$ of a SARS-CoV-2 variant, this is unlikely to be an important driver the transmission dynamics during a selective sweep, especially whilst strong NPIs are in effect. Whilst we should still be vigilant of increased transmission in colder months, especially in the absence of NPIs, the major factors mediating the transmission dynamics of a new sweeping variant are more likely to be intrinsic differences between a new variant and previous variants, particularly factors relating to immune evasion. However, as vaccine-mediated immunity wanes through time and NPIs are relaxed worldwide, we suggest that there is the possibility that future variants could switch *back* to being strongly influenced by climate. This may disproportionately impact the spread of new variants in colder environments, where further decreases in temperature may drive comparatively greater increases in transmission, whereas under generally warmer temperatures, small increases or decreases in temperature may matter little for changes in transmission.

## Methods

To investigate the impacts of climate on SARS-CoV-2 variant transmission, we combine estimates of transmission rate (*R*) for the Alpha, Delta, and Omicron strains during their respective sweeps, with climate and non-climate predictor variables, and then model the effects of these predictors. All data processing and analyses were performed in R, version 4.1.2^20^.

Before modelling the impacts of climate, we must first extract the variance attributable to other sources of spatial and temporal variation in *R*, which may mask or enhance the apparent climate response if unaccounted for. For instance, population density is expected to influence SARS-CoV-2 transmission rates^4–6^, but varies spatially with climate in England (the highest population densities are found in the warmer South–East, cf. Fig. 1a). Climate can also co-vary temporally with other variables that influence transmission, such as changes in NPIs through time^6,30^ and thus these should be explicitly accounted for. Furthermore, there may be other unknown sources of spatial and temporal variation in *R* that should also be accounted for (*e.g.* socioeconomic effects).

Here, we extract known and unknown sources of non-climate related variation in *R*, leaving a residual “corrected *R*”, upon which we model the effects of climate variables for each of the sweeping SARS-CoV-2 variants.

### Epidemiological data collection

Positive PCR tests using the ThermoFisher TaqPath assay return diagnostic failure on the S-gene target for the Alpha SARS-CoV-2 variant. In comparison, the Delta variant returns a positive test on the S-gene target. Whilst many other variants can result in S-gene target failure (SGTF), by November 2020, the Alpha variant was responsible for almost all SGTF PCR tests in the UK^16^. Alpha was from then the dominant strain in the UK, until the Delta variant (S-gene positive) swept through from April 2021 and became dominant in May^13^. The wide usage of this diagnostic PCR test in the UK has allowed SGTF to be used as a good proxy to predict the proportion of tests attributable to the Alpha variant^16^. These test results can also be used to differentiate Alpha from Delta during Delta’s selective sweep through the UK^13^. Similarly, as Omicron is S-gene negative and its sweep occurred at a time when previously most cases in England were attributed to the S-gene positive Delta variant, these test results can be against used to partition Omicron cases from Delta cases during Omicron’s selective sweep^15^.

Weekly SARS-CoV-2 transmission rates ($R_t$; the time-varying reproduction number) for the sweeping variants in each of England’s 42 National Health Service “Sustainability and Transformation Plan” regions (STPs) were estimated using a semi-mechanistic epidemiological model combined with SGTF frequency data^16^. For the Alpha selective sweep, these transmission rates were taken directly from Volz et. al.^16^, for the Delta and Omicron sweeps these rates were calculated for the current study, using the same methodology and data source as Volz et. al.^16^. A joint model for transmission of the sweeping variant against all others was fitted for each of the 42 STPs. For example, during the Delta sweep the joint model assumes that Delta is transmitting against a back drop of Alpha (the major variant before the Delta sweep). This joint model enables us to link the the transmission of variants within a STP and hence is more robust than fitting individual rates for each variant. The fitting was performed using the R package Epidemia^31^. The data for Alpha begins in week 45 of 2020 (2nd November), as prior to this there were too few Alpha cases to reliably estimate the transmission rates across England. Similarly, the data for Delta begins in week 18 of 2021 (3rd May) and the data for Omicron begins in week 47 of 2021 as before these dates there were insufficient Delta and Omicron cases respectively for reliable *R* estimation. The estimates for Omicron cover a shorter time period (4 weeks) than Alpha and Delta (8 and 9 weeks respectively) as the speed at which Omicron spread through the population meant that by late December 2021, S-gene positivity no longer provided a reliable prediction for Delta^15^. Prior to analysis we excluded the STP region “Cornwall and the Isles of Scilly” from all datasets due to there being too few cases for reliable *R* estimation in many weeks in this region.

### Transmission predictors data collection

To assess the impacts of climate variables on SARS-CoV-2 Alpha and Delta variant transmission, we obtained daily estimates of temperature, specific humidity, relative humidity, UV radiation and precipitation averaged across STP areas from AREAdata^32^. AREAdata publishes spatial averages of climate variables^32^ based upon the CDS-ERA5 climate dataset^33^, a source widely used in SARS-CoV-2 climate-response studies^3,4,6,27^. The AREAdata processing pipeline is built upon the same code-base as used in a previous SARS-CoV-2 seasonality study^6^. We aggregated these daily climate estimates to weekly mean values, for analysis with our weekly *R* estimates.

Population density has previously been shown to be a factor in SARS-CoV-2 transmission rates across space^4–6^. We also acquired population densities for STP regions from AREAdata.

The greatest factor in SARS-CoV-2 transmission rates is expected to be the strength of NPIs in place^2–6,30^, even if vaccination rates are high^28^. Similar to previous studies, we used the regularly updated Oxford COVID-19 Government Response Tracker’s (OxGRT) “Stringency Index” (SI) as an estimate of NPI strength^5,22,30^. The stringency index is a metric combining containment and closure policy indicators with an indicator of public information campaigns to estimate the overall strictness of “lockdown” policies restricting peoples’ movements and behaviour^22^. As with the climate data, these SI measures were aggregated to weekly means. The OxGRT data does not provide metrics specific to STP areas, so these data are single measures for the whole of England. By focusing on a single country, England, we are able to eliminate the large amount of cross-country variation in the effectiveness of these intervention strategies^34^.

Vaccinations had not yet been rolled out when the Alpha variant began to sweep through England, however by the time Delta arose, a vaccination program was well underway and much of the population was already fully vaccinated^28^ (*i.e.* had received two doses of the Pfizer/BioNTech or Oxford/AstraZeneca vaccinations). This vaccine driven immunity must therefore also be considered when modelling factors influencing transmission during the Delta variant sweep. We acquired daily vaccination data from the UK Government’s COVID-19 dashboard^35^. These are not available for STP regions, so we acquired these data for smaller spatial regions (lower-tier local authorities; LTLAs) and then aggregated these to STP regions based on shapefiles for STPs and LTLAs acquired from the UK’s Office for National Statistics portal^36^. We divide the number of fully vaccinated individuals by the total population of each region, to get the proportion of population vaccinated, which we use in our analysis.

### Regression analysis of environmentally driven transmission

In order to investigate the effects of climate variables on the transmission rates of the Alpha and Delta SARS-CoV-2 variants during their respective selective sweeps, we perform regression in a two-step process. We fit these models using least-squares regression. First, we regress the variant transmission rates ($R_{t,Alpha}$, and $R_{t,Delta}$, the time-varying reproduction numbers specific to each variant) against the known non-climate predictors of *R*, accounting for spatial variation and fluctuating temporal variation. For Alpha we account for NPI stringency, population density, STP areas and time (week), using:

1 $$\begin{aligned} \begin{aligned} R_{t,Alpha} = a + b_1 (\text {SI}) + b_2 (\log _{10}(\text {Pop density})) + b_3 (\text {Week}) + b_4 (\text {Week}^2) + \\ b_5 (\text {Week}^3) + b_{6...47} (\text {STP}_{1...41}) + \varepsilon , \end{aligned} \end{aligned}$$

where *a* is the intercept, $b_{1...47}$ are coefficients, SI is the stringency index, fluctuating weekly variation is modelled as a cubic function, STP is a categorical variable for area, and $\epsilon$ is an unmeasured residual error term. Exploratory investigations of the data showed oscillations in the transmission rate through time (see Fig. 2). Thus, to avoid spurious correlation with climate variables fluctuating through time, we used a cubic function to represent weekly variation in transmission. Population density was log-transformed as this logarithmic relationship with transmission rate has been observed in previous studies^5,6^.

For Delta, we additionally account for vaccinations, using the proportion of people fully vaccinated by area and week, with:

2 $$\begin{aligned} \begin{aligned} R_{t,Delta} = a + b_1 \text {(SI)} + b_2 (\log _{10}(\text {Pop density})) + b_3 \text {(Vaccination rate)} + b_4 \text {(Week)} + \\ b_5 \text {(Week}^2) + b_6 \text {(Week}^3) + b_{7...48} (\text {STP}_{1...41}) + \varepsilon . \end{aligned} \end{aligned}$$

When Omicron swept through England, previous immunity had waned, but booster vaccinations restored vaccine effectiveness in the population^37^. For Omicron we therefore fitted the same model as for Delta (Eq. 2), but with the proportion of people having received a booster vaccination dose as the vaccination rate. Diagnostic plots for these linear model fits are shown in supplementary figures S5-S7.

Next, we take the residuals from these models as the “corrected $R_t$”, i.e. $R_t$ after correcting for sources of non-climate variation, and regress these against the climate variables (temperature, specific humidity, relative humidity, UV radiation and precipitation), *i.e.*:

3 $$\begin{aligned} \begin{aligned} \text {Corrected}\ R_t = a + b_1 (\text {Temperature}) + b_2 (\text {Specific Humidity}) + \\ b_3 (\text {Relative Humidity}) + b_4 (\text {UV}) + b_5 (\text {Precipitation}) + \varepsilon . \end{aligned} \end{aligned}$$

This two-stage process was chosen to be conservative with regard to the estimation of climate effect, and to ensure that correlations between climate and other drivers of transmission would not affect our results. By statistically accounting for all terms other than climate first, including null terms ($b_{3...47}$ in Eq. 1 and $b_{4...48}$ in Eq. 2) for space and time, we ensure that expected, null dynamics (in each region an initial rise in cases and then a tailing off) would not spuriously be associated with climate effects. This approach has proved effective in previous studies^38^.

In all models, continuous variables are first scaled and centered by subtracting the mean and dividing by 2 standard deviations^39^ and therefore can be directly compared to understand the relative importance of each variable. Previous work has suggested that SARS-CoV-2 transmission responses to temperature may be non-linear^4,5,40^. To account for this, based on visualisation of our data we additionally fit an asymptotic model for temperature versus corrected $R_t$, as:

4 $$\begin{aligned} \text {Corrected}\ R_t = Asym + (R_0 - Asym) \cdot e^{(-e^{lrc} \cdot temperature)} + \varepsilon , \end{aligned}$$

where *Asym* represents the horizontal asymptote (*i.e.* corrected $R_t$ at very large values of temperature), $R_0$ is corrected $R_t$ when temperature is $0^{\circ }$C and *lrc* is the log-transformed rate constant. We compare the asymptotic model to a simple linear model of the form:

5 $$\begin{aligned} \text {Corrected}\ R_t = a + b (\text {Temperature}) + \varepsilon , \end{aligned}$$

using a likelihood-ratio test to determine the best fitting response-type. We additionally compare the model goodness of fit by AIC.

### Supplementary Information

Supplementary Information.

## Data Availability

The data and code generated for the current study are available in our GitHub repository: https://github.com/smithtp/covid-alpha-delta.
