# Supplementary material for: Differential responses of SARS-CoV-2 variants to environmental drivers during their selective sweeps
Source: Sci Rep. 2024 Jun 10;14:13326. doi: 10.1038/s41598-024-64044-1 (PMC11164892; doi:10.1038/s41598-024-64044-1)
Supplement: Supplementary file 1 — Supplementary Information. [file 41598_2024_64044_MOESM1_ESM.pdf]

Supplementary material for: Differential responses of SARS-CoV-2 variants to environmental drivers during their selective sweeps

Contents

Regression model results **1**

Collinearities between climate variables **3**

Linear regression model for alpha variant **6**

Model diagnostic plots **6**

Regression model results

Table S1: **Climate drove variation in R_t during Alpha variant sweep.** Here, $r^2 = 25\%$, $F_{5,322} = 21.53$, and $p < 0.001$. Coefficient estimates are when predictors are scaled and centered by subtracting the mean and dividing by 2 standard deviations (see Gelman (1)) and thus the absolute values measure the relative importance of each variable. After removal of non-climate-related variation in transmission rates, we find that temperature, UV and precipitation all have significant linear effects, but that temperature is the comparatively strongest predictor. Here, * = $p < 0.05$.

	Coefficient	Std. Error	t value	p value
(Intercept)	0.18	0.03	5.69	< 0.001*
Temperature	-1.13	0.40	-2.81	0.005*
Specific Humidity	0.35	0.34	1.03	0.304
Relative Humidity	-0.07	0.06	-1.13	0.260
UV	0.25	0.05	4.98	< 0.001*
Precipitation	0.16	0.05	3.20	0.002*

Table S2: **Climate had no significant effect on R_t during the Delta variant sweep.** Here, $r^2 = 0.8\%$, $F_{5,363} = 0.55$, and $p = 0.739$. Coefficient estimates are when predictors are scaled and centered. Once non-climate-related variation has been removed, we find no significant effects of any of the climate variables on transmission during the Delta sweep.

	Coefficient	Std. Error	t value	p value
(Intercept)	0.00	0.01	0.00	1.00
Temperature	-0.12	0.21	-0.56	0.57
Specific Humidity	0.10	0.21	0.49	0.63
Relative Humidity	0.02	0.06	0.23	0.82
UV	0.02	0.04	0.48	0.63
Precipitation	-0.02	0.03	-0.62	0.53

Table S3: **Climate had no significant effect on R_t during the Omicron variant sweep.** Here, $r^2 = 2.3\%$, $F_{5,154} = 0.74$, and $p = 0.594$. Coefficient estimates are when predictors are scaled and centered. Once non-climate-related variation has been removed, we find no significant effects of any of the climate variables on transmission during the Omicron sweep.

	Coefficient	Std. Error	t value	p value
(Intercept)	0.00	0.06	0.00	1.00
Temperature	1.28	0.85	1.53	0.13
Specific Humidity	-1.98	1.15	-1.72	0.09
Relative Humidity	0.64	0.52	1.23	0.22
UV	-0.10	0.33	-0.31	0.76
Precipitation	-0.37	0.24	-1.58	0.12

Table S4: **Asymptotic versus linear functions of the temperature response.** Here, we assess the goodness of fit of our linear and asymptotic models for the response of R_t to temperature. For each variant, we give the AIC and log-likelihood for both models. We also provide the p-value for a likelihood ratio test, assessing whether the asymptotic model is a better fit than the linear model. * = $p < 0.05$, *i.e.* where the asymptotic model is a significantly better fit. The asymptotic model is a better fit to the R_t data for the Alpha variant, both by AIC and by the likelihood ratio test. R_t showed no association with temperature for Delta or Omicron, but the goodness of fit for these models are included here for completeness.

Variant	Linear AIC	Asymptotic AIC	Linear log-likelihood	Asymptotic log-likelihood	likelihood ratio test p-value
Alpha	300.71	279.37	-147.35	-135.68	< 0.001*
Delta	-66.29	295.66	36.15	-143.83	1
Omicron	389.30	395.91	-191.65	-193.95	1

Collinearities between climate variables

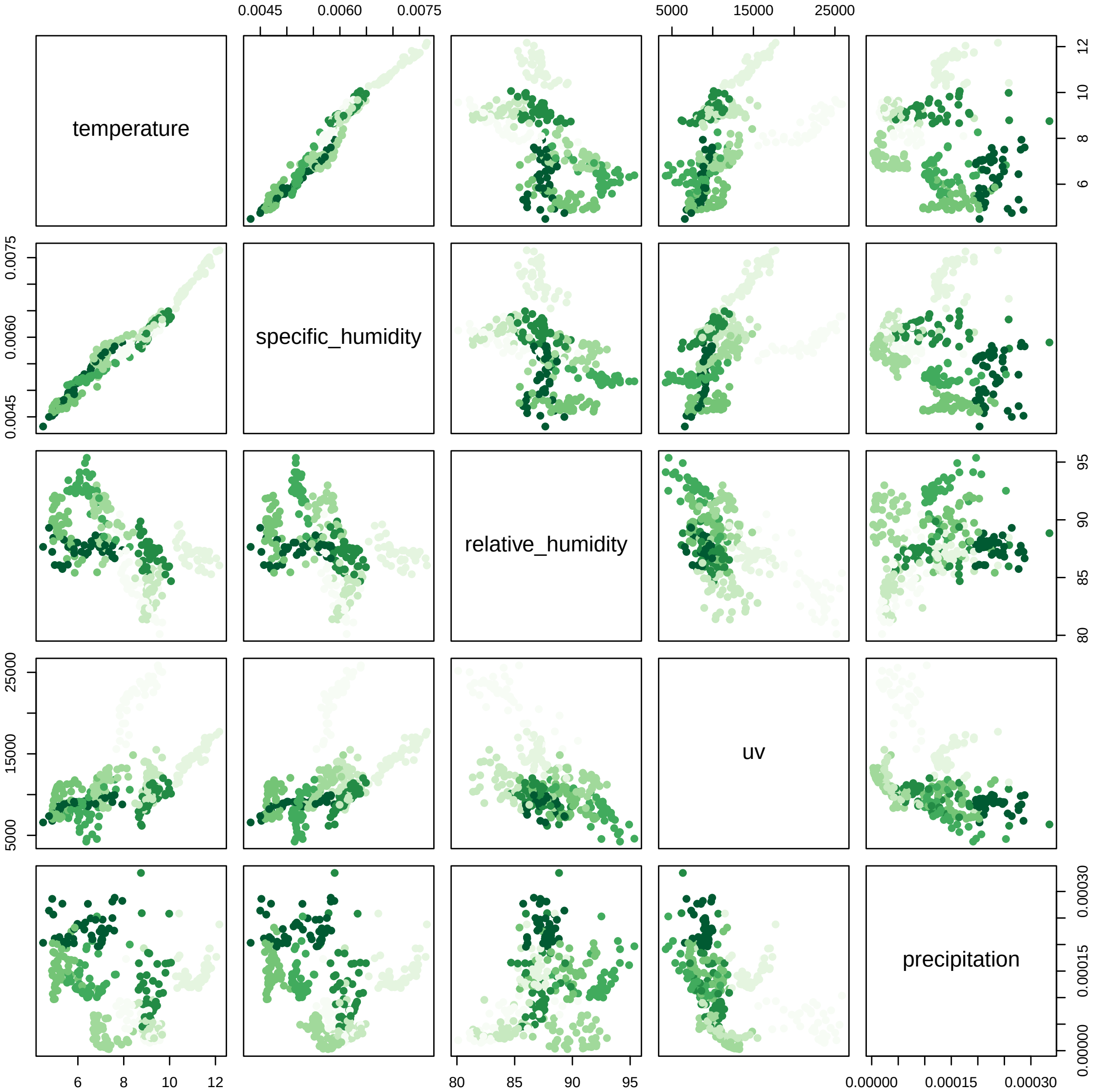

Figure S1: **Collinearities of climate variables during Alpha sweep.** Pair-wise correlations between the climate variables used in our models (temperature, specific humidity, relative humidity, UV and precipitation), between weeks 45 (light green) and 52 (dark green) of 2020 (the Alpha variant sweep).

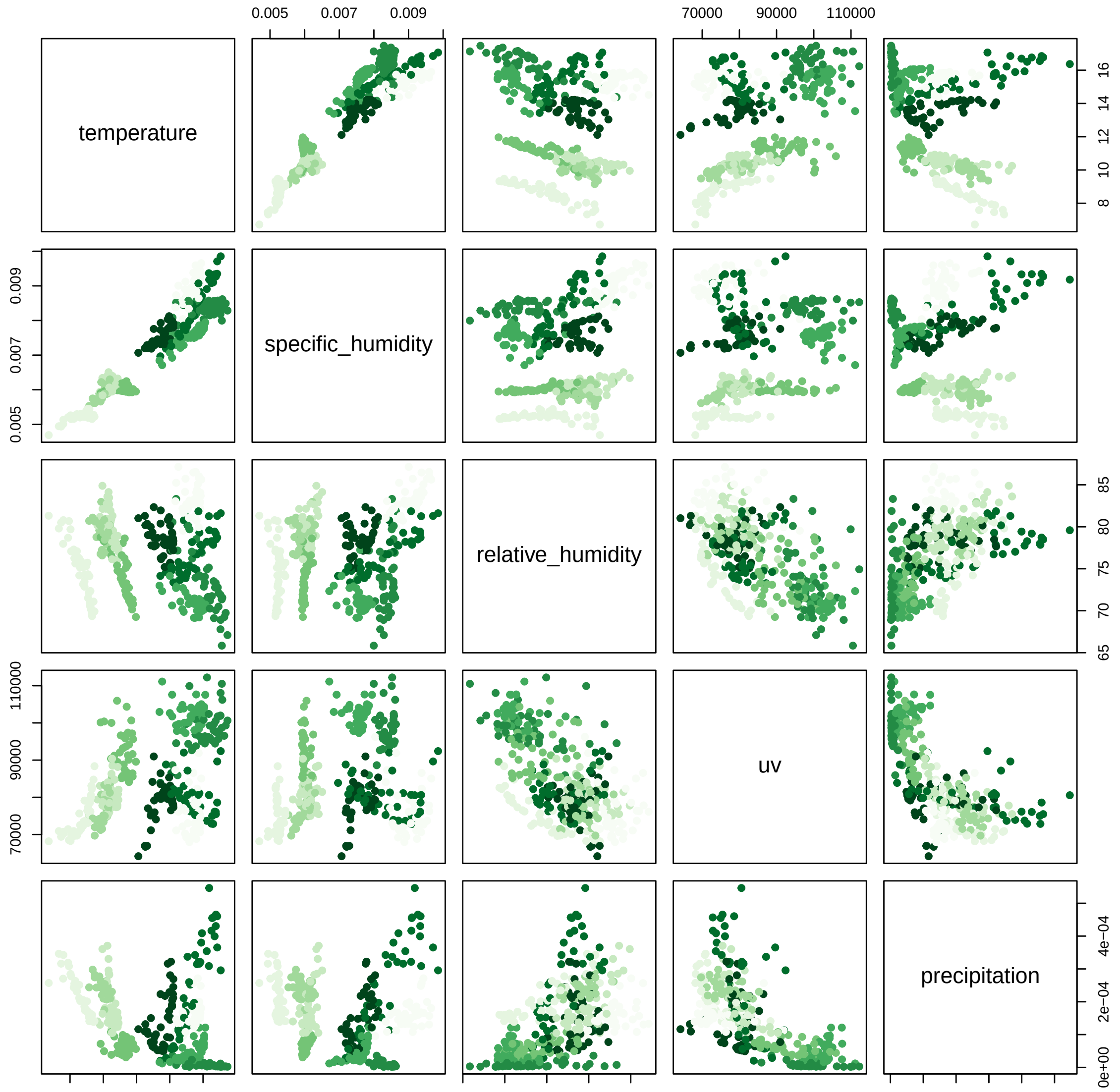

Figure S2: **Collinearities of climate variables during Delta sweep.** Pairwise correlations of climate variables between weeks 18 (light) and 26 (dark) of 2021.

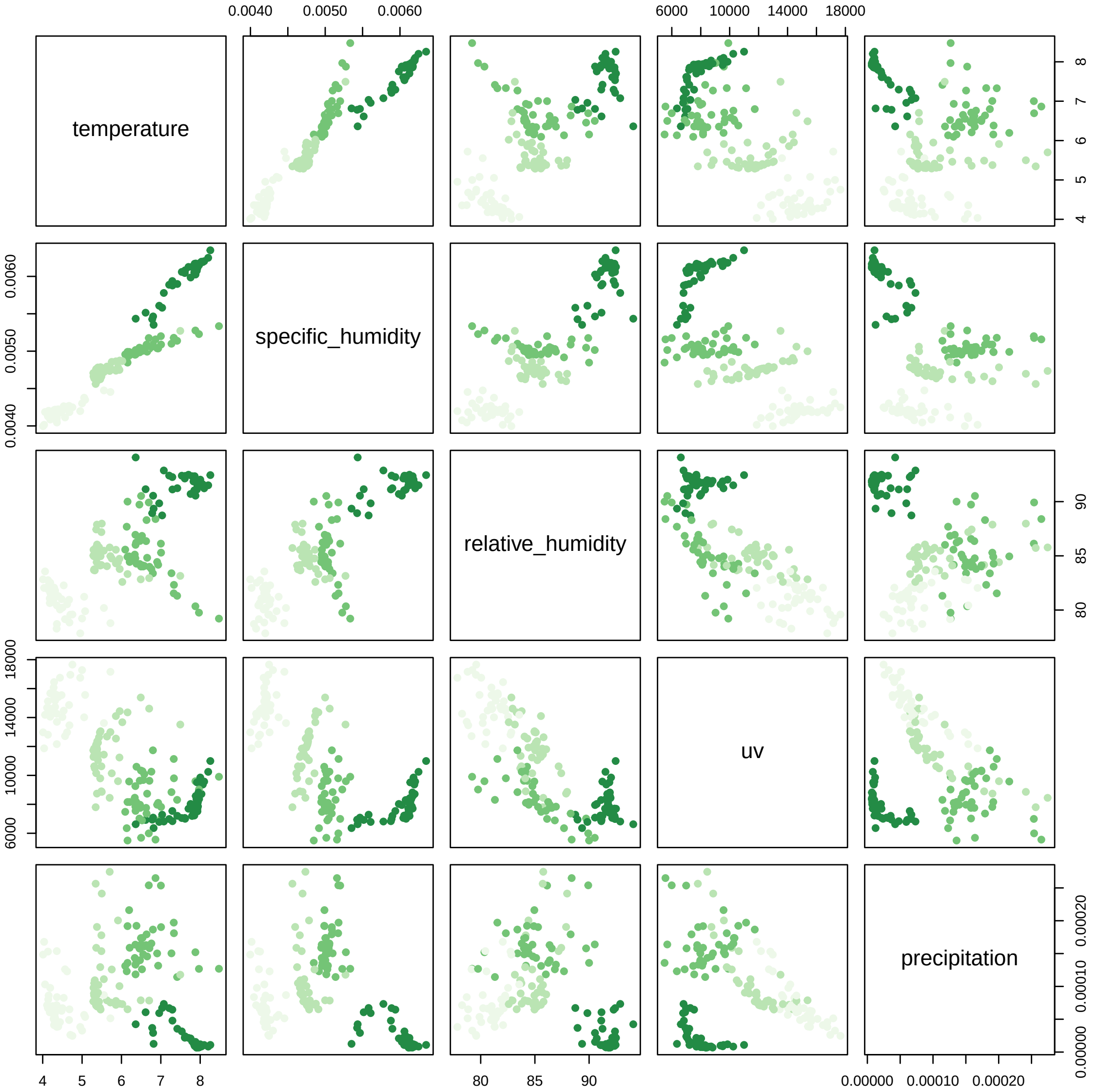

Figure S3: **Collinearities of climate variables during Omicron sweep.** Pairwise correlations of climate variables between weeks 47 (light) and 50 (dark) of 2021.

Linear regression model for alpha variant

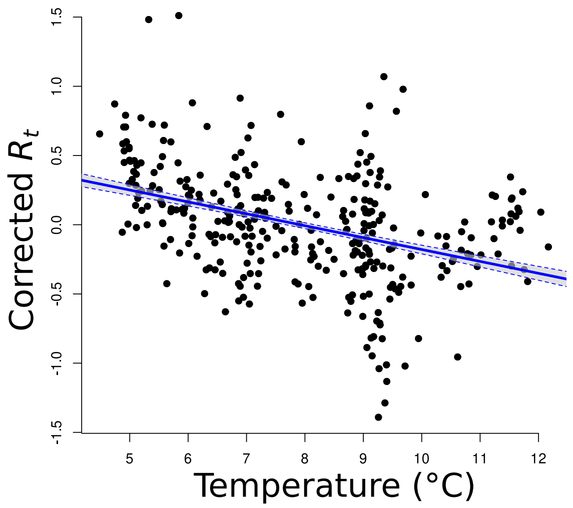

Figure S4: **Alpha variant linear model fit.** Here we plot the residuals from a linear regression of R_t versus all of the non-climate spatial and temporal predictors for the Alpha variant (termed "corrected R_t"), against temperature. A linear model provides a significant fit (intercept = 0.68, slope = -0.086, $p < 0.001$, $r^2 = 15.4\%$), however an asymptotic model is preferred to the linear model by a likelihood ratio test (see main text).

Model diagnostic plots

Figures S5-S7 show diagnostic plots from the linear model fits to the non-climate variables, for each variant respectively.

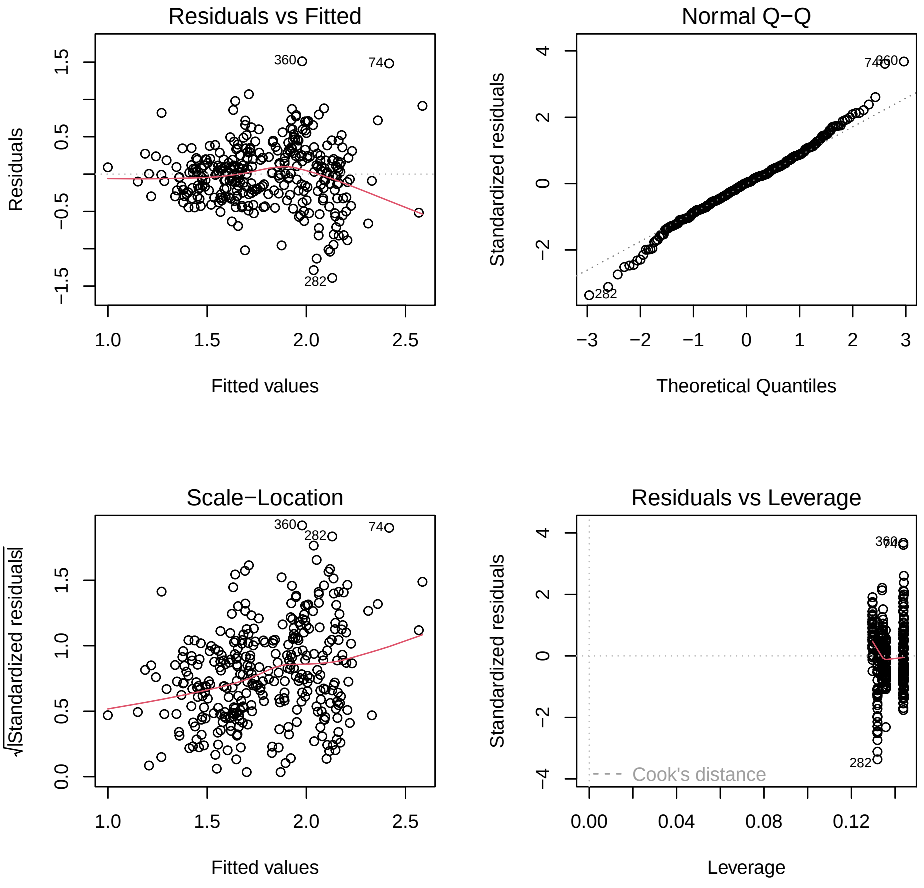

Figure S5: **Alpha variant model diagnostic plots.**

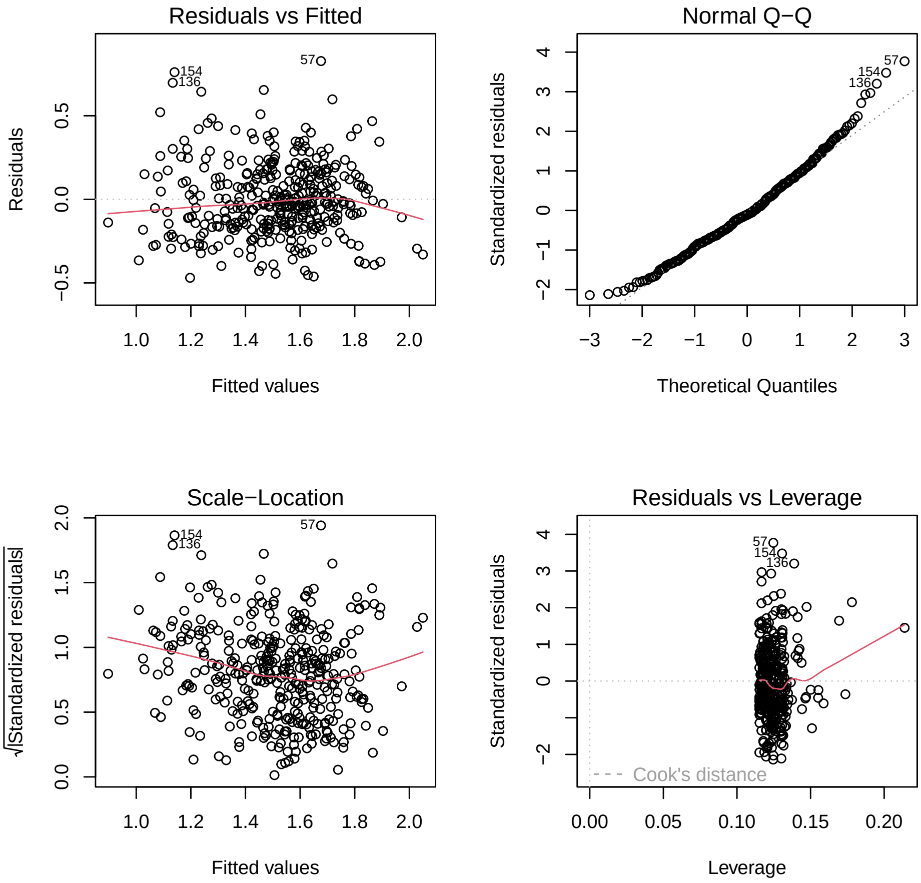

Figure S6: **Delta variant model diagnostic plots.**

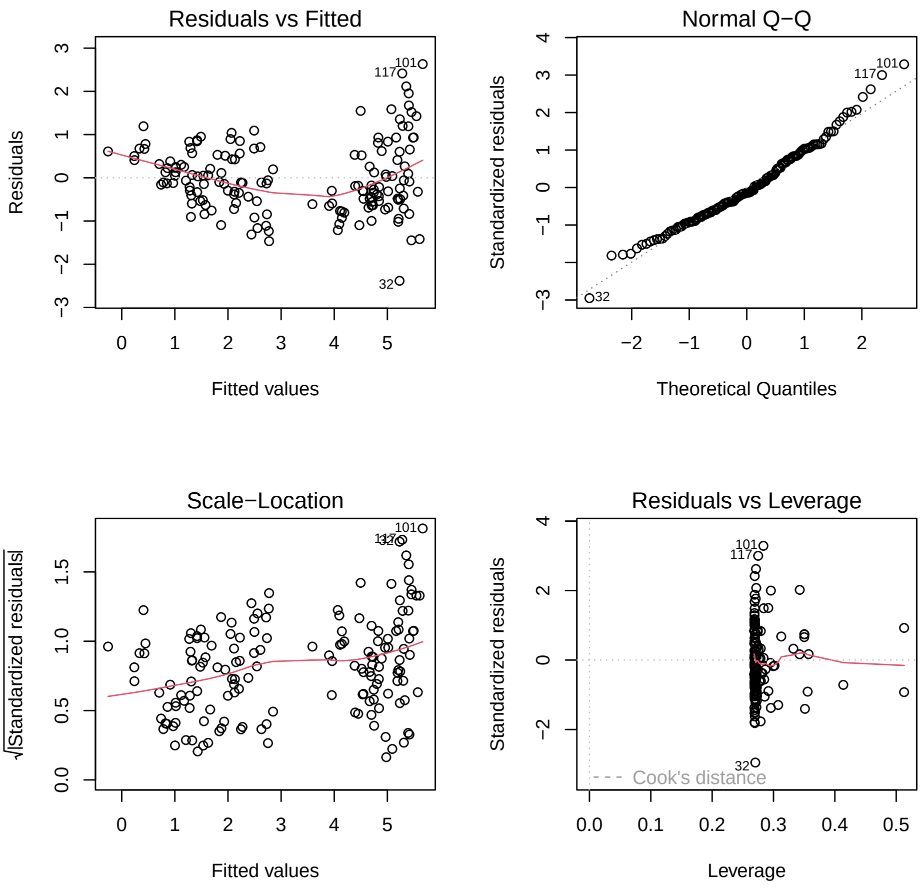

Figure S7: **Omicron variant model diagnostic plots.**

References

[1] Gelman A. Scaling regression inputs by dividing by two standard deviations. Statistics in Medicine. 2008;27:2865-73.
